# LPP3, LPA and self-generated chemotactic gradients in biomedical science

**DOI:** 10.1080/19420889.2017.1398870

**Published:** 2017-12-14

**Authors:** Olivia Susanto, Robert H. Insall

**Affiliations:** CR-UK Beatson Institute, Switchback Road, Glasgow G61 1BD, UK

**Keywords:** cell biology, chemotaxis, memes, metastasis, lipid breakdown, lipid signalling

## Abstract

Chemotaxis is a major driver of cancer spread, but in most cases we do not know where gradients of attractant come from. In the case of melanoma, chemotaxis to LPA is an important driver of metastasis, and the gradients are made by the tumour cells themselves, by locally breaking down ambient LPA. We have now made a general assay for self-generated chemotaxis, and used it to show that the enzyme LPP3 is responsible for breaking down LPA and thus creating the gradients. Further analysis shows LPP3 is important in several invasion assays, in particular 3D ones in which cells spread outwards through matrix. The new assays will illuminate where physiological self-generated gradients occur; we believe they will be common throughout biology and pathology.

The ability of cells to migrate across relatively large distances underpins many developmental, immunological and pathological processes. Chemotaxis, the migration of cells in response to a gradient of a chemical stimulus, is often a driver of cell migration in these circumstances. However, there are relatively few conditions in which a stable environmentally imposed chemotactic gradient could be maintained over a long distance *in vivo*. This would require not only a source producing high concentrations of the attractant at one site to compensate for the distance over which the gradient must be established, but also an attractant sink at the other end of the gradient to absorb it. Thus, we have hypothesised that **self-generated gradients** – in which cells create their own chemotactic gradients while they migrate – are an efficient way to direct cell migration over longer distances *in vivo*.^1^ Indeed, various groups have recently demonstrated self-generated gradients in cell migration in multiple biological processes. These include primordium migration in developing zebrafish and cellular organisation in the lymph nodes and spleen.^2–4^ These studies demonstrated the formation of the self-generated gradients using reporters targeting key proteins involved in the process. We have shown that self-generated gradients are used by metastatic melanoma cells to promote their own spread,^5^ using a new method to detect self-generated chemotactic gradients when no precise pathway is known.^6^

## Self-generated LPA gradients in melanoma metastasis

Lysophosphatidic acid (LPA) is a key signalling molecule present ubiquitously in the serum and tissues of the body, which can stimulate migration of several cell types, including melanoma cells.^5,7–9^ Interestingly, melanoma cells are not only highly chemotactic towards LPA, but also efficiently break exogenous LPA down into inactive products.^5,6^ Thus melanoma cells possess the two major requirements to produce self-generated gradients – the ability to respond to the chemoattractant and also remove it locally from the environment. Accordingly, when melanoma cells are placed in an environment of uniform serum/LPA, after a delay they are able to break it down, form their own local chemotactic gradients and thus migrate away from areas of high cell density. The lipid phosphatase LPP3 (also known as PPAP2B) is critical for melanoma cell-mediated LPA breakdown, and is consequently a central player in formation of self-generated LPA gradients.^6^ While LPP3-depleted cells are still chemotactic towards LPA when an external gradient is imposed, they can no longer produce self-generated gradients to stimulate cell migration in uniform serum environments.

## A general assay for the formation of self-generated gradients

**Figure 1.**
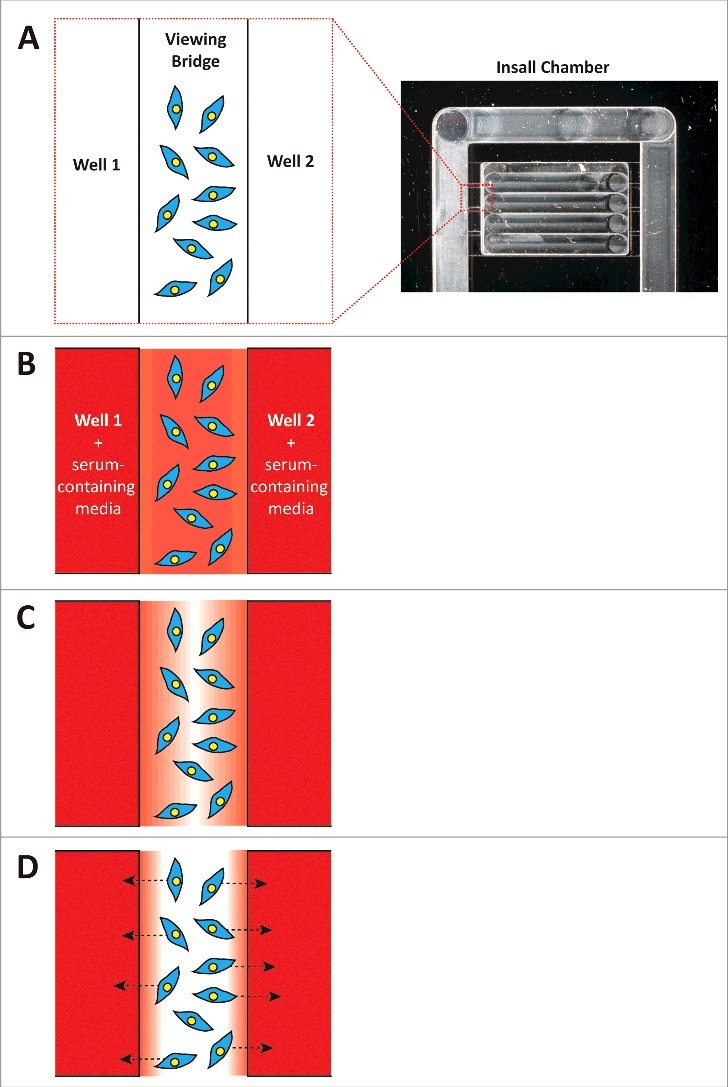
(A) Cells are seeded on coverslips, which are placed atop an Insall chemotaxis chamber as described in.^13^ The cells are visualised on the viewing bridges, which are positioned between two wells. (B) Serum-containing media is added to both wells, and it diffuses across the viewing bridge. (C) Over time, the cells on the viewing bridge degrade the LPA in the serum. (D) Eventually, the LPA on the bridge has been depleted and the cells begin to migrate towards the sources of more LPA (i.e. the serum in the wells). The cells on the left side of the bridge tend to migrate towards the well on the left, while the cells on the right tend to migrate to the well on the right, resulting in a distinct bi-directional pattern of chemotaxis.

In order to determine the role of LPP3 in melanoma cell migration, we developed an assay to measure the formation of self-generated gradients. As it is difficult to directly visualise gradients that accurately represent biologically active vs. inactive LPA, we utilised a functional readout of self-generated gradient formation (i.e. cell chemotaxis) (Fig. 1). This assay could specifically determine whether cells were responding to self-generated gradients rather than migrating randomly, as a serum-free environment did not produce a similar effect.^6^ Given the simplicity of the assay, it is ideal for testing whether different cell types are able to produce and respond to self-generated chemotactic gradients, in the presence of either serum or specific chemoattractant targets.

## Self-generated gradients in other systems

We found that self-generated gradients were also important in 2D and 3D invasion assays where matrigel was overlaid to mimic the extracellular environment. This suggests that the slight restriction of chemoattractant flow in the presence of matrigel also facilitates the formation of self-generated gradients, and should be taken into account when using such assays. Similarly, the migration of *Dictyostelium* under agarose due to self-generated gradients is enabled by the restriction of chemoattractant diffusion by the agarose layer.^10^ Above all, chemotaxis should be considered a contributor to invasion assays even if no gradients are provided – cells may be making their own gradients as they invade.

We believe that self-generated gradients – caused either by local breakdown of attractants, as described above, or by other mechanisms such as secreted autorepellents^11^ and even local breakdown of morphogens^12^ – will turn out to be extremely widespread throughout biology. Their characteristics of self-organisation and robustness make them able to function under conditions where simpler imposed gradients are broken – and robust mechanisms are strongly favoured during evolution.

## Parameters in motility assays

The concept of self-generated gradients changes the way we envisage directed cell migration. Even in assays where gradients are provided externally, cells can modulate their responses by local gradient sharpening. It is usually presumed that a higher concentration of chemoattractant at one end of a path will produce a steeper gradient, and consequently better chemotaxis; clearly this is not the case. When observing chemotaxis of metastatic melanoma or *Dictyostelium* cells, imposing external gradients with very high concentrations of chemoattractant is actually detrimental to the efficiency of chemotaxis.^6,10^ Cells migrated more efficiently towards a chemoattractant source when its local concentration does not saturate the receptors. When excessively high attractant concentrations are used, self-generated chemotaxis can still occur, but with a delay caused by the requirement to break down excess chemoattractant to levels at which a gradient can be generated.^6^ When cells cannot break down the chemoattractant, chemotaxis cannot proceed in areas close to the source of high chemoattractant.^10^ Thus, the combined effects of chemoattractant breakdown and receptor saturation must always be kept in mind when studying cell chemotaxis, whether the gradients are wholly self-generated or formed from external sources and sinks.
